# On the Employment of Local Injections in Neuralgia, Paralysis, and Other Affections

**Published:** 1866-05

**Authors:** 


### On the Employment of Local Injections in Neuralgia, Paralysis, and other Affections.

Prof. Courtey, of Montpelier, has published a note on the efficacy of local injections of strychnia in the treatment of paralysis of the facial nerve. He injected a few drops of a solution of this alkaloid along the course of the facial nerve, between its exit by the stylo-mastoid foramen and its passage to the neck of the condyle of the lower jaw. The injection was repeated every two or three days, and three injections at the least, and six at the most, sufficed to remove entirely, in the space of from ten to fifteen days, every trace of paralysis in all the muscles of the face. The patients were a man aged fifty-six, a lady of twenty-five, and a young woman of twenty-two. In all three cases the cure was complete. M. Courty has also recorded a case of paralysis which lasted for a year, and had been ineffectually treated by various remedies, but which was cured by a few injections of strychnia, performed over the inferior extremity of the spinal cord. M. Luton, of Rhiems, has also called attention to the use of local injections in various maladies. He has successively employed a more or less concentrated solution of nitrate of silver in 12 cases of sciatic neuralgia, 2 cases of intercostal neuralgia, 3 of coxal neuralgia, etc. M. Luton has also mentioned a curious case of suborbital neuralgia removed by three injections of salt water. He has three times employed injections of tincture of iodine in parenchymatous goitres; one case was cured, the other two were still under treatment at the date of the report. The applications of which this new plan is susceptible are very numerous, and may include the use of bichloride of mercury, arsenious acid, sulphate of copper, sulphate of zinc, and any other irritating substance which acts in the interior of the tissues in the same manner as one which is applied on their surface.—*British and Foreign Medico-Chirurgical Review, July*, 1864, *p.* 241.

